# Limited Predictability of Traumatic Intracranial Hemorrhage from Routine Pre-CT Clinical Variables in Older Adults with Low-Energy Falls: A Systematic Benchmarking Study in a Retrospective Bicentric Cohort

**DOI:** 10.3390/diagnostics16172840

**Published:** 2026-09-03

**Authors:** Robert Stahl, Anna Theresa Stüber, Rebecca Wania, Michael Ingrisch, Maryam Ostadi Ataabadi, Marco Öchsner, Robert Forbrig, Christoph G. Trumm, Thomas Liebig, Wolfgang Böcker, Vera Pedersen

**Affiliations:** 1 Institute for Diagnostic and Interventional Neuroradiology, LMU University Hospital, LMU Munich, Marchioninistr. 15, 81377 Munich, Germany; 2 Department of Radiology, LMU University Hospital, LMU Munich, Marchioninistr. 15, 81377 Munich, Germany; 3 Department of Orthopaedics and Trauma Surgery, Klinikum Dritter Orden, Menzinger Str. 44, 80638 Munich, Germany; 4 Radiologie Augsburg Friedberg ÜBAG, Hermanstraße 15, 86150 Augsburg, Germany; 5 Department of Orthopaedics and Trauma Surgery, Musculoskeletal University Center Munich (MUM), LMU University Hospital, LMU Munich, Marchioninistr. 15, 81377 Munich, Germanyvera.pedersen@med.uni-muenchen.de (V.P.)

**Keywords:** traumatic intracranial hemorrhage, machine learning, low-energy falls, older adults, emergency department, benchmarking, class imbalance, computed tomography

## Abstract

**Background/Objectives**: Traumatic intracranial hemorrhage (tICH) in older adults following low-energy falls (LEF) represents a common yet diagnostically challenging condition in the emergency department (ED), where predicting injury prior to computed tomography (CT) remains difficult. Machine learning (ML) has been proposed to support CT decision-making, but its feasibility using routinely available pre-CT clinical variables in this specific population remains unclear. This study presents a systematic exploratory benchmarking of ML pipeline configurations for pre-CT tICH prediction in a well-defined retrospective cohort of older emergency patients following LEF. **Methods**: We performed a secondary analysis from a retrospective observational bicentric study from two university hospital EDs, including 2250 patients aged ≥65 years presenting after an LEF and undergoing cranial CT. Clinical data were extracted manually from electronic health records (EHRs). Eighteen pre-CT clinical features retrieved from electronic health records were selected based on routine availability and ≤10% missingness. Overall, 1224 valid ML pipeline configurations, combining nine classification algorithms, six imputation strategies, four class-balancing approaches, and optional hyperparameter tuning, were evaluated using 10-fold stratified cross-validation on a training set. The 20 highest-ranked configurations by cross-validation AUC were then assessed on a previously inspected exploratory hold-out test set (*n* = 563); training-derived rule-out operating points were evaluable for 17 of these 20, as three tuned SVM configurations lacked stored out-of-fold predictions. **Results**: tICH prevalence was 7.0% (*n* = 158). Across the 20 highest-ranked configurations, hold-out AUC ranged from 0.517 to 0.585, with Matthews correlation coefficient near zero and balanced accuracy of approximately 50% throughout, indicating differences in operating point rather than in discriminative ability. Some of these top-ranked pipelines reached higher cross-validation AUC (up to 0.679) but detected no cases at the default 0.5 threshold—an effect of the decision threshold under class imbalance rather than of the models’ rank-order discrimination, which was itself limited (hold-out AUC of 0.517–0.585). **Conclusions**: Despite comprehensive exploratory benchmarking across 1224 ML pipelines, routinely available pre-CT clinical features did not provide sufficient discriminatory signal to develop a clinically useful tICH prediction model in this cohort of CT-imaged older adults following LEF. These findings indicate that none of the evaluated configurations produced clinically adequate performance; this near-chance result persisted across all pipelines and most plausibly reflects a combination of limited feature signal, low outcome prevalence, and a sample size below the level required for reliable model development at this event rate. Future studies should target substantially larger prospective multicenter cohorts and evaluate additional feature domains, including structured clinical examination findings, point-of-care biomarkers, and imaging features.

## 1. Introduction

Head trauma in older adults is a frequent and clinically important problem in emergency departments (EDs). Even seemingly minor head trauma can result in traumatic intracranial hemorrhage (tICH), particularly in patients with age-related cerebral atrophy, fragility of bridging veins, multimorbidity, and widespread use of antithrombotic medication [1]. As populations age, emergency physicians are increasingly confronted with older patients after falls, in whom the decision to perform cranial computed tomography (CT) is often difficult [2,3].

Low-energy falls (LEF), commonly defined as falls from standing height or lower, are the predominant mechanism of head injury in older adults and represent the primary context in which this risk materializes [4]. Substantial rates of tICH have been documented even after LEF, ranging from approximately 5 to 7% in ED-based cohorts [4,5] to over 11% in patients without focal neurological findings [6], yet these events are frequently perceived as minor and the severity of injury underestimated [7]. This creates a diagnostic dilemma in the ED: timely CT imaging is essential to avoid missed hemorrhage, yet liberal scanning strategies lead to substantial overuse of imaging, resource consumption, and radiation exposure [8,9].

Current clinical decision rules, such as the Canadian CT Head Rule [10], the New Orleans Criteria [11] and the recent guidelines of the National Institute for Health and Care Excellence (NICE, [12]), have improved the evaluation of patients with minor head injury [7,13]. However, these rules were developed in broadly mixed populations and were designed to achieve very high sensitivity, often at the expense of specificity. In older patients, this approach may result in particularly low specificity and therefore a high proportion of unnecessary CT scans. The Falls Decision Rule (FDR) [14] and its recent external validation [15] represent the most pertinent non-ML benchmark for this clinical problem and are recently discussed in this context. More individualized risk prediction tools may therefore better reflect the risk profile of older patients after low-energy trauma.

Machine learning (ML) has been proposed as a potential approach to augmenting CT decision-making by integrating multiple bedside variables simultaneously and generating individualized probability estimates for tICH [16,17]. In this specific population, prior work has examined individual predictors [4,5,18], but has not systematically benchmarked the broader space of routinely available pre-CT clinical variables using ML. Whether this routine feature space contains sufficient discriminatory signal to support such models in older adults after LEF therefore remains to be established through systematic evaluation, which is the aim of the present study.

However, applying ML in emergency medicine also poses important methodological challenges. Severe class imbalance, missing data, heterogeneous patient populations, and the need for rigorous external validation all complicate model development and interpretation [19]. More broadly speaking, caution is advised, when the claims of clinical impact are outpacing the scientific rigor needed to support them, to avoid premature clinical implementation [20]. Accordingly, transparent benchmarking of competing ML pipelines is essential to identify robust strategies and to define the practical limits of prediction in real-world ED data.

This study presents a systematic exploratory benchmarking of more than 1200 ML pipeline configurations for the prediction of tICH in older adults following LEF using pre-CT clinical data from two tertiary care university hospital EDs in Switzerland and Germany. Rather than developing or validating a clinical prediction model, we systematically benchmark the tICH-discrimination performance of routinely available EHR-based features across a comprehensive set of ML pipelines in this well-characterized but sample-size-constrained real-world dataset. In doing so, we aim to provide transparent, hypothesis-generating evidence on the feasibility of ML-based tICH prediction from routine clinical data.

## 2. Materials and Methods

### 2.1. Study Population and Feature Selection

Data originate from a bicentric, retrospective observational study conducted in the EDs of the University Hospital in Basel (Switzerland) and the LMU University Hospital, LMU Munich (Germany).

The study adhered to the Declaration of Helsinki and STROBE guidelines [21]. Ethics approval was granted by the Ethics Committee of Northwest and Central Switzerland (EKNZ: 2017-01078) and the Ethics Committee of LMU Munich (EK LMU 17-217). Informed consent was waived given the retrospective design.

**Figure 1:**
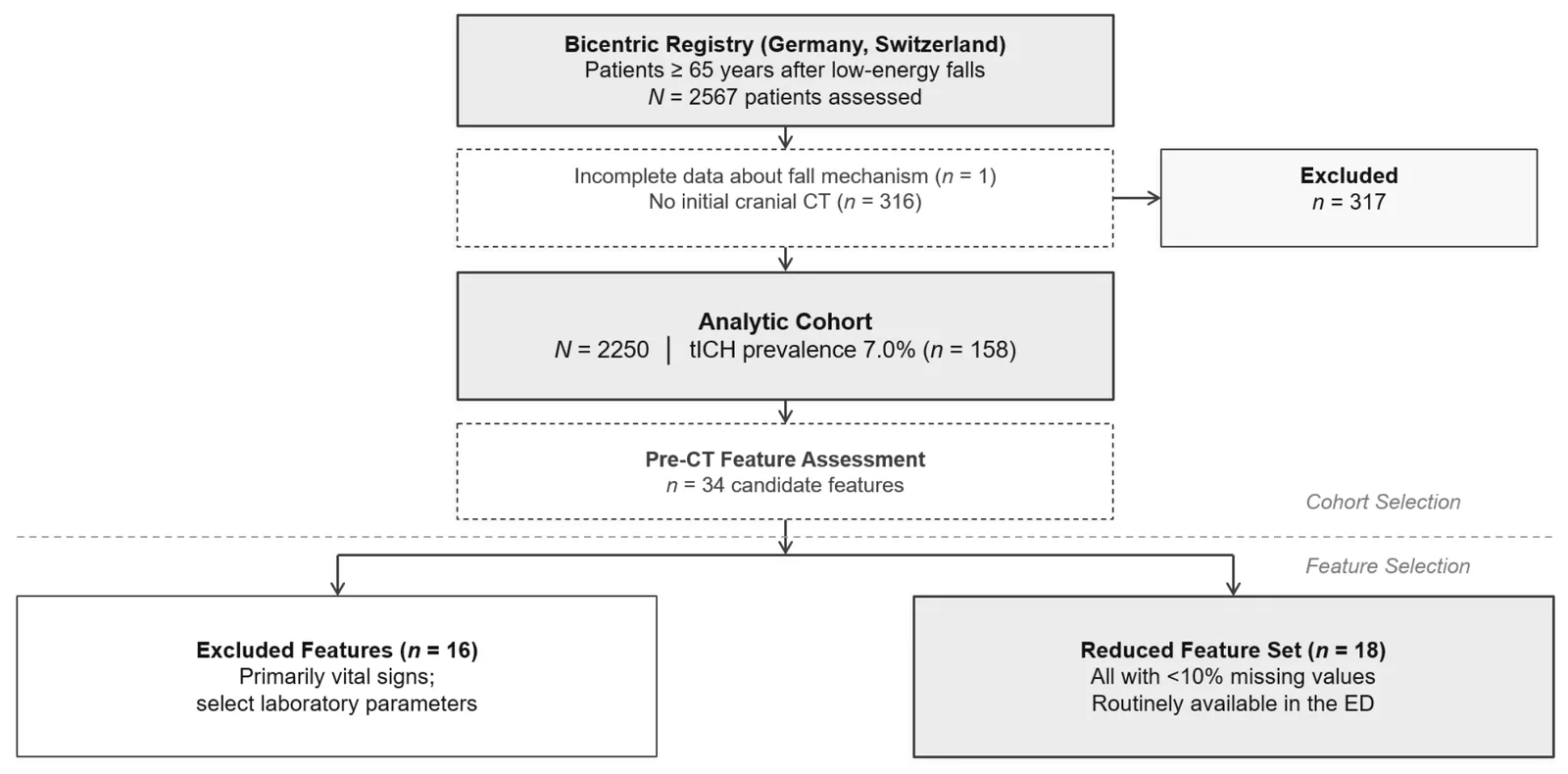
Flowchart illustrating cohort selection and feature reduction. ED = emergency department; tICH = traumatic intracranial hemorrhage; CT = computed tomography; *N* = total cohort; *n* = subset.

Patients aged ≥65 years presenting to either ED between 1 January 2016 and 31 December 2016 following a documented LEF with cranial CT within 48 h were eligible [4]. A subset of the present cohort, in numbers the 951 patients from the LMU Munich site (January–December 2016), was previously included in the single-center (LMU Munich) study by Wania et al. [18] (*n* = 2687, September 2014–December 2016). This previous study addressed the diagnostic value of the protein S100b biomarker as predictor for tICH in elderly persons with LEF, and used conventional regression and ROC analysis. The protein S100b biomarker is not part of the routinely documented pre-CT feature set analyzed here, and no results from that study are reused. LEF was defined per ICD-10 codes as previously described [22]: fall from standing height (W00, W01, W03, W04, W18), fall from low furniture (W05–W08), or fall from ≤1 m (W10) within 7 days before presentation. Patients were excluded if they presented via resuscitation room or trauma team activation, were referred with preceding imaging, managed by a fast-track process, presented ≥8 days after the fall, re-presented for the same incident, or received CT solely distal to knee or elbow. The initial cohort comprised 2567 patients [4]; after excluding 317 patients (1 with incomplete data about fall mechanism, 316 without initial cranial CT), the analytic cohort comprised 2250 patients (Figure 1).

Clinical data extraction from electronic health records (EHRs) was performed as described previously [4]. High data fidelity was ensured by manual extraction of clinical data from the full EHRs by two independent, trained observers; all discrepancies were identified and resolved by a third independent observer. No automated data extraction procedures were employed. In this context, a missing value indicates that the respective feature was not documented in the patient’s EHR at the time of the ED encounter.

The primary outcome was traumatic intracranial hemorrhage (tICH) confirmed on cranial CT, encompassing subdural hematoma, epidural hematoma, subarachnoid hemorrhage, intracerebral hemorrhage, and parenchymal contusions. tICH status was taken from routine clinical radiology reports; all examinations were interpreted by board-certified radiologists or neuroradiologists, with off-hours resident reads validated by a specialist by the following day. Reports were unambiguous regarding the presence or absence of tICH, and no study-specific re-review or blinded double reading was performed. tICH prevalence in the analytic cohort was 7.0% (*n* = 158).

We initially identified 34 pre-CT clinical features as candidate predictors; baseline characteristics of the study cohort across all features are presented in Table S1. To reflect a realistic ED setting, we focused on routinely documented variables and excluded features with ≥10% missing values, yielding a reduced set of 18 predictors (Figure 1). This missingness threshold was applied as a pragmatic criterion reflecting clinical documentation consistency, acknowledging that features with high missingness rates—in the context of manual EHR extraction—cannot be reliably imputed and would introduce substantial uncertainty into model training. The retained features included demographics (age, sex), triage parameters (Glasgow Coma Scale [GCS], Emergency Severity Index [ESI]), mechanism of fall, clinical findings (loss of consciousness, supraclavicular injury signs), anticoagulation and antiplatelet status (vitamin K antagonists [VKA], non-vitamin K oral anticoagulants [NOAC], single/dual antiplatelet therapy, low-molecular-weight heparin [LMWH], no anticoagulation or antiplatelet therapy, anticoagulation/antiplatelet status unknown), polymedication, and laboratory values (serum sodium, platelet count, prothrombin time [Quick]). These 18 pre-CT clinical features represent a routinely available feature set, defined by clinical availability and a pragmatic missingness threshold applied independently of outcome associations, rather than by formal prespecification. They reflect information documented at triage or during initial clinical assessment in the ED. Retained features are highlighted in bold in Table S1.

### 2.2. Machine Learning Pipelines

**Figure 2:**
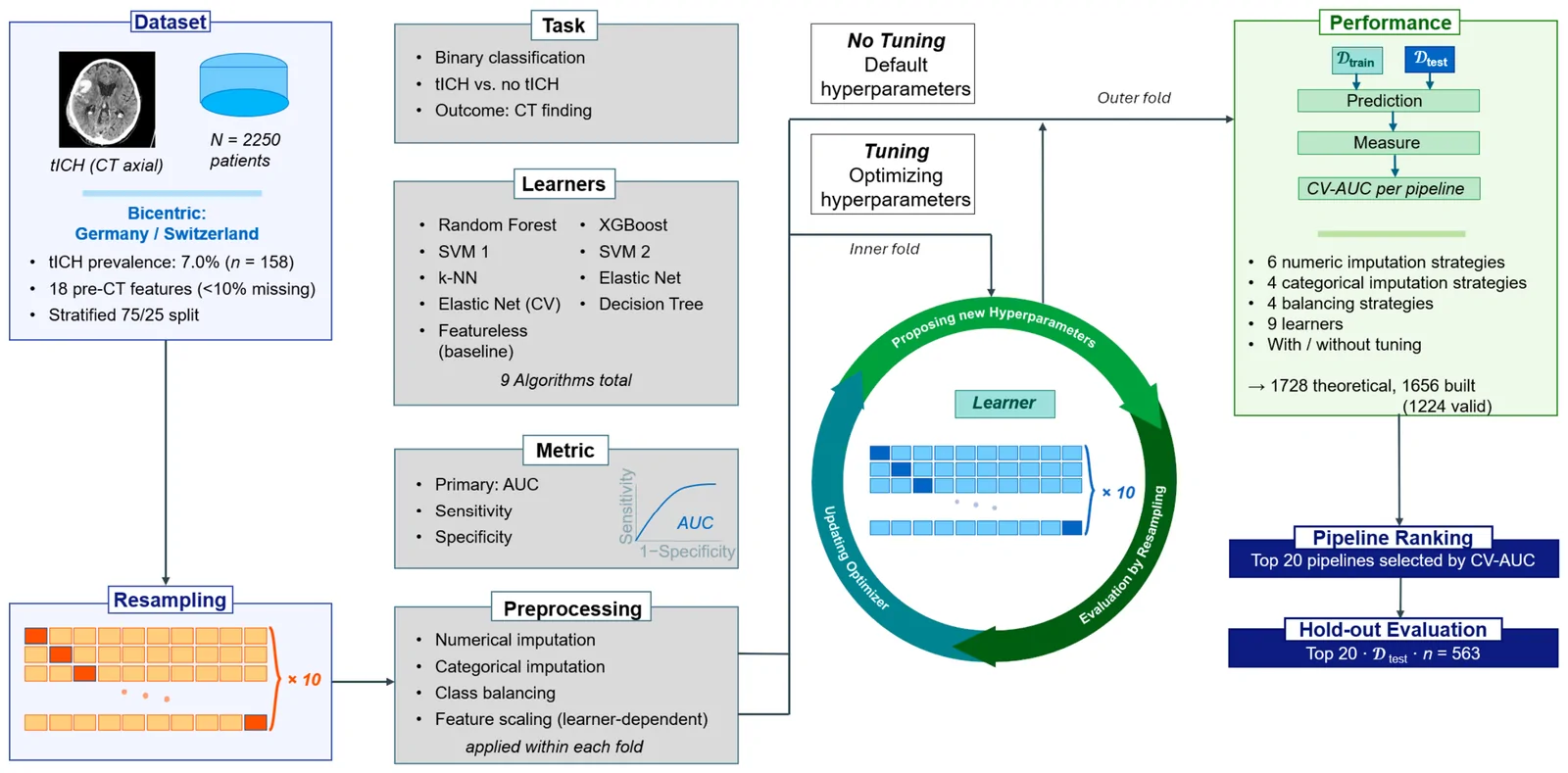
Schematic of the machine learning benchmarking framework. The analytic cohort (*N* = 2250) was split into training (75%, *n* = 1687) and hold-out test sets (25%, *n* = 563) via stratified random sampling. Each pipeline combined a preprocessing sequence (numerical imputation, categorical imputation, class balancing, and learner-dependent feature scaling) with one of nine classification algorithms, with or without hyperparameter tuning, yielding 1728 theoretically possible configurations, of which 1656 were constructed and 1224 completed successfully. All valid configurations were evaluated by 10-fold stratified cross-validation (CV-AUC). The 20 highest-ranked configurations by cross-validation AUC were retrained on the full training set and evaluated on the hold-out test set. AUC = area under the receiver operating characteristic curve; CV = cross-validation; k-NN = k-nearest neighbors; SMOTE = Synthetic Minority Oversampling Technique; SVM = support vector machine; CT = computed tomography; tICH = traumatic intracranial hemorrhage. Colors group the stages of the workflow: blue = data and resampling, gray = pipeline components, green = hyperparameter tuning and performance estimation, dark blue = ranking and hold-out evaluation. In the resampling grids, each row represents one cross-validation iteration; light cells denote training folds and the dark cell denotes the held-out fold (outer loop, orange; inner tuning loop, blue). $D_{\mathrm{train}}$ and $D_{\mathrm{test}}$ denote the training and hold-out sets.

Machine learning pipelines were implemented using the mlr3 ecosystem [23], including mlr3 (core framework, version 1.2.0), mlr3pipelines (modular pipeline construction, version 0.9.0), mlr3tuning (hyperparameter optimization, version 1.4.0), and mlr3learners (algorithm implementations, version 0.12.0). All terminology regarding pipeline components and algorithm designations follows mlr3 conventions. An overview of the complete benchmarking framework is provided in Figure 2; the following sections describe each component in detail.

Each pipeline configuration consisted of four sequentially applied components: a numerical imputation strategy, a categorical imputation strategy, a class-balancing strategy, and a classification algorithm, optionally combined with hyperparameter tuning. Combining all component options yielded 1728 theoretical configurations; after exclusion of 72 incompatible combinations, 1656 configurations were submitted for benchmarking, of which 1224 were valid and evaluated (see Section 3.2 for details).

#### 2.2.1. Data Preprocessing

Preprocessing was applied as an integral part of each pipeline configuration and executed sequentially prior to model fitting. Missing values were addressed through systematic imputation, with six strategies evaluated for numerical features (no imputation, median, mean, histogram-based, out-of-range, and random sample imputation) and four for categorical features (no imputation, mode, out-of-range, and random sample imputation). Out-of-range imputation replaces missing values with a value outside the observed feature range, allowing algorithms to learn missingness as an implicit pattern. Algorithms unable to handle missing values natively required complete imputation and were therefore only valid in combination with non-missing imputation strategies.

Given the class imbalance in the analytic cohort (tICH prevalence 7.0%), four balancing strategies were evaluated: no balancing, random oversampling of the minority class, random undersampling of the majority class, and Synthetic Minority Oversampling Technique (SMOTE) [24]. SMOTE generates synthetic minority-class samples by interpolating between existing minority observations and requires complete prior imputation. Categorical features were one-hot encoded prior to interpolation, and standard SMOTE was applied to the resulting numerical representation; the categorical-specific variant SMOTE-NC was not used. Interpolation on encoded indicator variables can yield fractional values, which the downstream tree-based and regularized learners accommodate. SMOTE was thus evaluated as one of four balancing strategies rather than as a sole choice; cost-sensitive learning through class weighting represents a further alternative that was not examined here and could be considered in future work. Balancing was applied exclusively to training data. Feature scaling and one-hot encoding were applied in a learner-dependent manner. All preprocessing parameters were estimated exclusively on training folds and applied without re-estimation to test folds, ensuring no information from test observations influenced model training.

#### 2.2.2. Machine Learning Algorithms

Nine classification algorithms representing diverse learning paradigms were evaluated, including ensemble methods, kernel-based classifiers, regularized regression, and instance-based learning [25,26,27,28,29,30,31,32,33]. The selection aimed to cover a broad spectrum of algorithmic approaches rather than optimizing for a single method, enabling a comprehensive comparison across different inductive biases and sensitivity to class imbalance. A featureless baseline classifier—predicting the majority class for all observations—was included as a lower performance bound; any useful model must substantially outperform this baseline. Brief descriptions of each algorithm are provided in Supplementary Text S1.

#### 2.2.3. Hyperparameter Tuning

Hyperparameter search spaces were defined according to the empirically derived recommendations of Probst et al. [34], who systematically evaluated hyperparameter importance and optimal tuning ranges across 38 datasets. Tuning employed 30 random search iterations optimizing CV-AUC within an inner 10-fold cross-validation loop. The ‘no tuning’ condition used algorithm-specific defaults and was evaluated directly within the outer cross-validation loop without an inner resampling step. Full search space specifications and algorithm-specific default values are provided in Table S2. The distinction between the tuned and untuned evaluation paths within the cross-validation framework is illustrated in Supplementary Figure S1.

#### 2.2.4. Model Evaluation

All valid pipeline configurations were evaluated by 10-fold stratified cross-validation on the training set, yielding a cross-validation AUC (CV-AUC; the primary, threshold-independent discrimination metric), sensitivity and specificity per pipeline. The 20 configurations with the highest CV-AUC were carried forward; no single pipeline was selected, and all 20 are reported on an equal footing. Each was retrained on the full training set and evaluated on the hold-out test set (*n* = 563). Because this partition had already been inspected during earlier exploratory analysis, it is described throughout as a post hoc exploratory test set; the corresponding estimates are reported for descriptive purposes only and should not be interpreted as independent, unbiased, or as constituting validation.

The area under the precision–recall curve (AUPRC) and calibration were additionally assessed. Given the low tICH prevalence (7.0%) and the rule-out context, two operating points were defined for each configuration entirely within the training data—the highest probability thresholds reaching at least 95% and at least 97.9% cross-validation sensitivity (the latter matching the 97.9% sensitivity reported for the Falls Decision Rule; that rule, however, targets clinically important intracranial hemorrhage, whereas the present outcome comprises any tICH detected on CT)—and applied to the hold-out predictions. Because the three tuned SVM configurations among the top 20 (SVM 2; ranks 15–17) had their tuning truncated by a per-fit time limit and their out-of-fold predictions were not stored, training-derived operating points could not be computed for them; the operating-point (rule-out) analyses therefore apply to 17 of the 20 top-ranked configurations, whereas threshold-independent discrimination and calibration are reported for all 20. At each operating point, sensitivity, specificity, positive and negative predictive value, F1 score, Matthews correlation coefficient, balanced accuracy, the full confusion matrix and the proportion of potentially avoided CT examinations are reported (95% confidence intervals where applicable).

As a sensitivity analysis, the entire procedure was repeated after excluding three laboratory predictors (serum sodium, platelet count, prothrombin time) whose timing relative to the CT decision cannot be guaranteed (15-feature model; Supplementary Table S5).

### 2.3. Statistical Analysis and Software

All analyses were performed in R version 4.3.3 (R Core Team, 2024. R: A Language and Environment for Statistical Computing. R Foundation for Statistical Computing, Vienna, Austria. https://www.R-project.org/, accessed on 15 September 2025). Machine learning pipelines were implemented using the mlr3 ecosystem as described in Section 2.2. All stochastic steps—the train–test split, cross-validation folds, model fitting, hyperparameter tuning and SMOTE—were governed by a single fixed random seed (20251002), and exact package versions were pinned with renv (version 1.1.5). The analysis code, the fixed seed, the renv lock file containing the complete package version list, and the derived result tables are deposited in Zenodo (https://doi.org/10.5281/zenodo.21996510; see Data Availability).

Baseline characteristics were compared between tICH and no-tICH groups using Welch’s *t*-test for normally distributed continuous variables, Wilcoxon rank-sum test for non-normally distributed continuous and ordinal variables, and Fisher’s exact test for categorical variables. Effect sizes were reported as mean difference with 95% CI (Welch’s formula), odds ratio with 95% CI (Fisher’s exact), and Hodges–Lehmann estimator with 95% CI (Wilcoxon). Statistical significance was set at α = 0.05. Because 34 univariate comparisons were performed, *p*-values were additionally interpreted under Benjamini–Hochberg false-discovery-rate control (*q* < 0.05).

The full pipeline benchmarking computations were performed on a dedicated server (16 virtual CPU cores, AMD EPYC Milan, 128 GB RAM) in October–November 2025; all preceding development and exploratory analyses were conducted on standard personal computers.

### 2.4. Artificial Intelligence Assistance

Claude (Anthropic, San Francisco, CA, USA; https://claude.ai, accessed on 21 August 2026) was used to assist in writing and debugging R code for the machine learning pipeline implementation, to support complex figure generation, and for language editing. Elicit (Elicit, Inc., Oakland, CA, USA; https://elicit.com, accessed on 27 July 2026) was used to support literature search and retrieval. All AI-assisted output was reviewed and verified by the authors.

## 3. Results

### 3.1. Study Population and Baseline Characteristics

In univariate group comparisons, seven features of the final feature set were nominally associated with tICH (*p* < 0.05): GCS, platelet count, loss of consciousness, ESI, male sex, LMWH use and unknown anticoagulation/antiplatelet status. After Benjamini–Hochberg correction for the 34 comparisons, significance persisted for GCS (lower in the tICH group, *p* < 0.001), platelet count (211.2 vs. 236.6 × 10^3^/µL, *p* < 0.001) and loss of consciousness (13.9% vs. 7.0%, OR 2.16, 95% CI: 1.27–3.52, *p* = 0.004); serum albumin—a descriptive laboratory variable not included in the model owing to high missingness—was the only further variable to survive correction (Table S1); the associations with ESI (*p* = 0.011), male sex (*p* = 0.020), LMWH use (*p* = 0.023) and unknown anticoagulation/antiplatelet status (*p* = 0.040) did not survive correction and may reflect chance.

### 3.2. Systematic Pipeline Benchmarking

Of 1728 theoretically possible pipeline configurations, 72 were excluded prior to execution due to incompatible component combinations, leaving 1656 configurations submitted for benchmarking. Of these, 432 were automatically skipped at runtime—288 due to algorithms requiring complete data combined with incomplete imputation strategies, and 144 due to SMOTE requiring complete prior imputation—leaving 1224 configurations that completed successfully. Benchmarking of these 1224 valid configurations required a total computation time of 10 days, 22 h, and 43 min.

### 3.3. Cross-Validation Pipeline Evaluation

**Figure 3:**
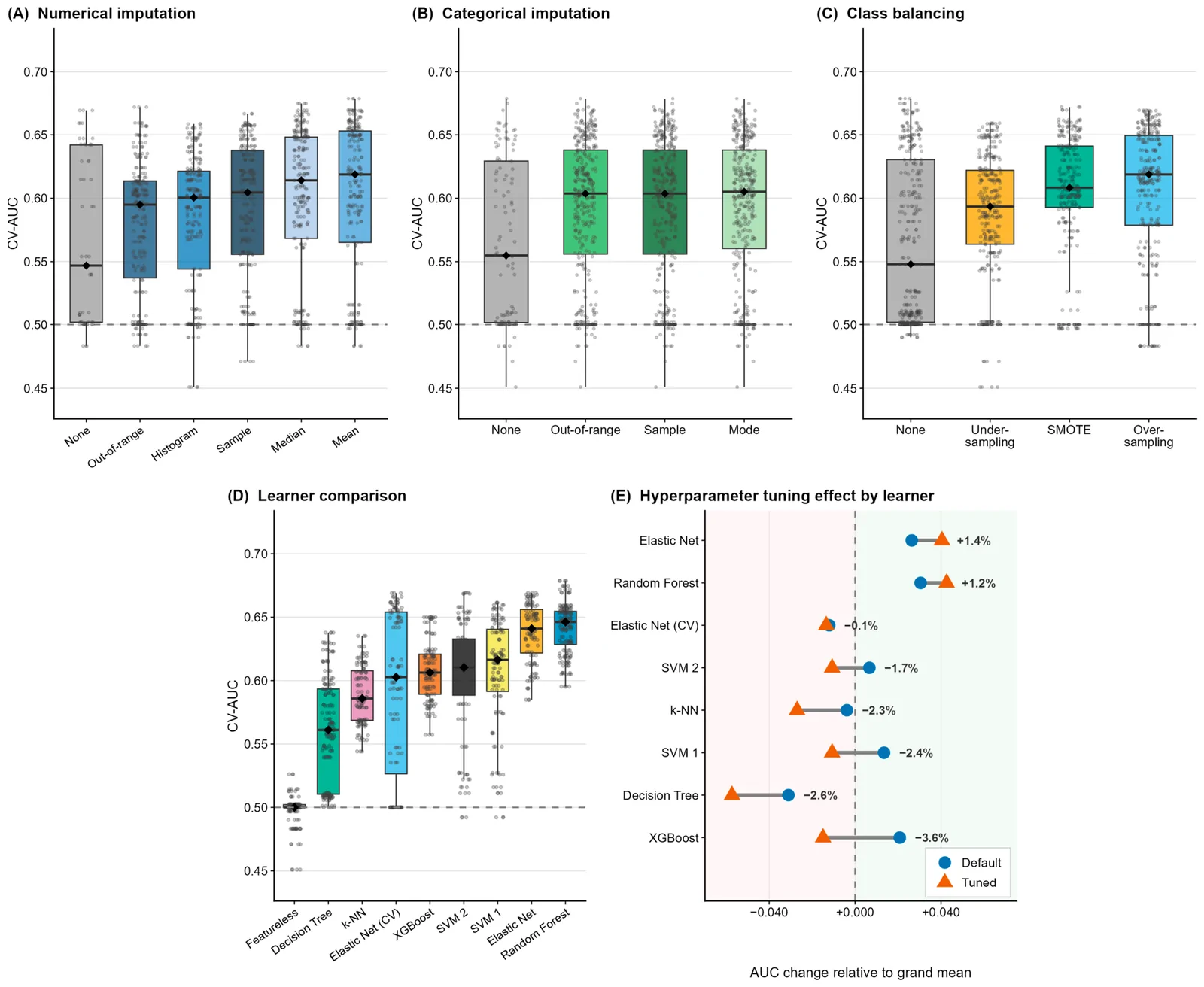
Cross-validation AUC (CV-AUC) across 1224 pipeline configurations, stratified by pipeline component. Boxplots show median, IQR, and 1.5 × IQR; the dashed line indicates chance-level performance (CV-AUC = 0.50). Categories are ordered by ascending median CV-AUC, with the reference category (no preprocessing applied) shown first. Panels: (**A**) numerical imputation, (**B**) categorical imputation, (**C**) class balancing, (**D**) classification algorithm, (**E**) hyperparameter tuning effect per learner, expressed as mean CV-AUC relative to the grand mean; the featureless baseline is not shown in panel (**E**), as it has no hyperparameters to tune. SVM = support vector machine; k-NN = k-nearest neighbors; SMOTE = Synthetic Minority Oversampling Technique. Panels show the marginal distribution of CV-AUC for each component, aggregated over all other pipeline components; as the grid is not a balanced factorial design and interactions are not modeled, differences between categories represent marginal patterns rather than isolated component effects.

Results of the systematic cross-validation benchmarking across all 1224 valid pipeline configurations are summarized in Figure 3.

These comparisons describe marginal patterns across the 1224 configurations rather than isolated effects of individual pipeline components: because the benchmarking grid is not a balanced factorial design and component interactions were not modeled, each panel reflects the marginal distribution of CV-AUC for one component aggregated over all others. Regarding numerical imputation, mean imputation achieved the highest median CV-AUC (0.619, IQR 0.565–0.653), followed by median imputation (0.614, IQR 0.568–0.648), while no imputation yielded the lowest performance (0.547, IQR 0.502–0.642; Figure 3A). For categorical imputation, mode, out-of-range, and sample imputation performed comparably (median CV-AUC 0.604–0.605), with no imputation again lowest (0.555; Figure 3B). Among class-balancing strategies, random oversampling achieved the highest median CV-AUC (0.619, IQR 0.579–0.649), followed by SMOTE (0.608) and undersampling (0.593), while pipelines without balancing yielded the lowest performance (0.548, IQR 0.502–0.630; Figure 3C). Random Forest achieved the highest median CV-AUC across learners (0.646, IQR 0.628–0.655), followed by Elastic Net (0.641); SVM variants, XGBoost, and Elastic Net (CV) showed intermediate performance (0.603–0.616), while k-NN (0.586) and Decision Tree (0.561) performed lowest, and the featureless baseline reached AUC = 0.500 as expected (Figure 3D). Hyperparameter tuning improved performance most for Elastic Net (+1.4%) and Random Forest (+1.2%), whereas tuning decreased CV-AUC for XGBoost (−3.6%), Decision Tree (−2.6%), SVM 1 (−2.4%), k-NN (−2.3%), and SVM 2 (−1.7%; Figure 3E).

### 3.4. Hold-Out Evaluation

**Figure 4:**
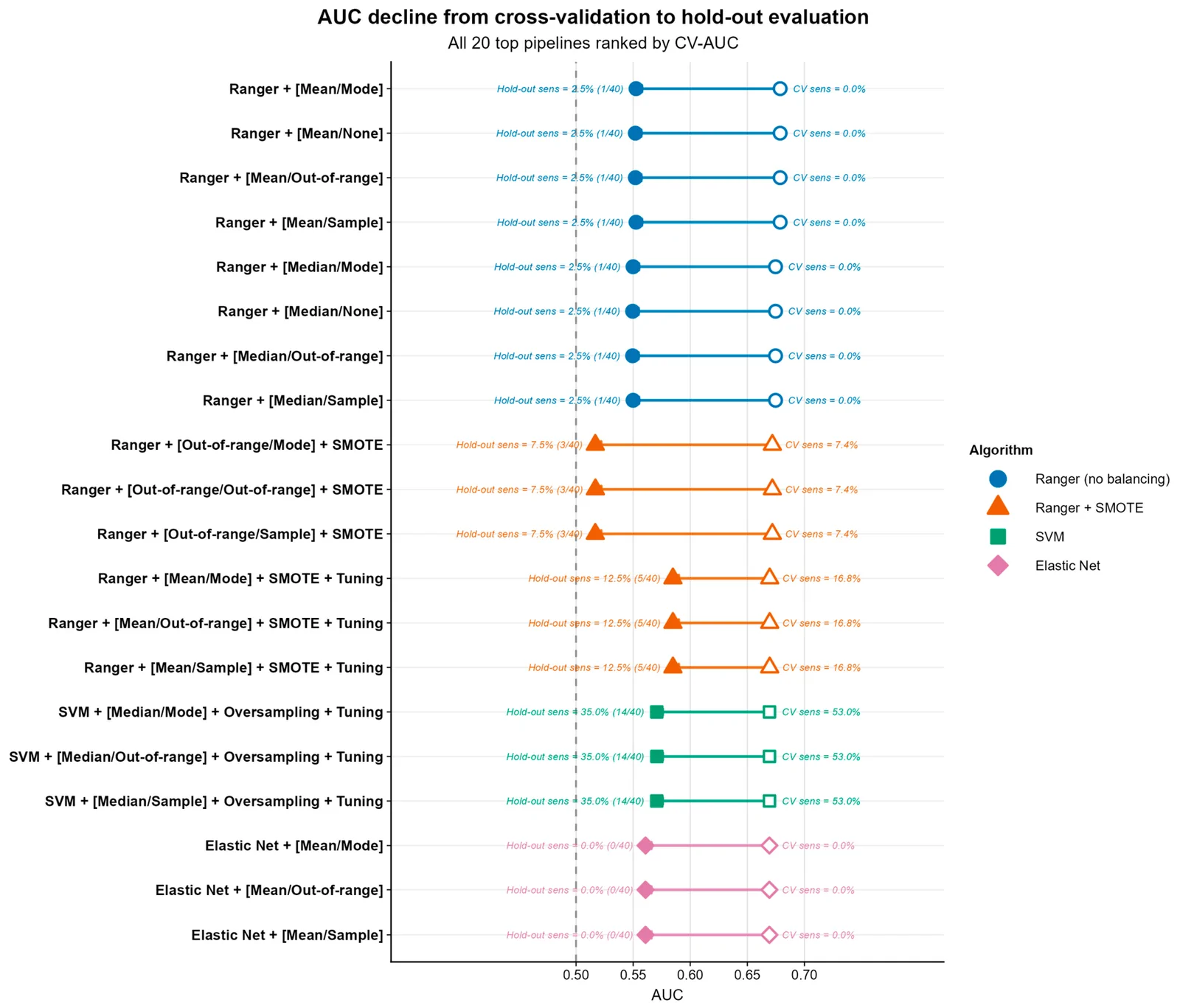
Cross-validation versus hold-out AUC for all 20 top pipeline configurations ranked by cross-validation AUC (CV-AUC). Each row represents one pipeline; open symbols indicate CV-AUC and filled symbols indicate hold-out AUC, connected by a horizontal line reflecting the CV-to-hold-out AUC decline. Hold-out sensitivity, with the number of correctly identified tICH cases, is annotated for each configuration. Symbols are colored by algorithm and class-balancing strategy. All pipelines cluster near AUC 0.50 on hold-out evaluation regardless of sensitivity, with hold-out balanced accuracy of 49.9–51.7% and Matthews correlation coefficient near zero across all configurations, indicating differences in operating point rather than in discriminative ability. The three tuned SVM pipelines (ranks 15–17) are exploratory, as hyperparameter search was truncated by a per-fit time limit; they lack stored out-of-fold predictions and are therefore excluded from the operating-point analyses, which apply to 17 of the 20 configurations. The dashed line marks the chance level (AUC 0.50).

Cross-validation and hold-out performance of the top 20 pipelines by CV-AUC are summarized in Figure 4.

Eleven of the top 20 configurations—the eight non-balancing Random Forest pipelines and the three Elastic Net pipelines—had a cross-validation sensitivity of 0% at the 0.5 threshold. Because AUC is threshold-independent, this reflects the naïve 0.5 threshold rather than a difference in discrimination, and all configurations are reported together. These configurations included the highest cross-validation AUC values overall (up to 0.679); at the naïve 0.5 threshold, however, all 11 had zero sensitivity, an artifact of that specific operating point rather than a lack of ranking ability. On the hold-out set, they identified at most one of 40 tICH cases (sensitivity ≤ 2.5%).

Among the configurations with non-zero cross-validation sensitivity, three pipelines shared the highest cross-validation AUC (0.672): all combined Random Forest, out-of-range numerical imputation, and SMOTE without tuning, differing only in categorical imputation strategy (mode, out-of-range, sample). These three were identical across all cross-validation metrics.

One example configuration is described here only to illustrate behavior at the naïve 0.5 threshold: the pipeline with the highest cross-validation AUC among those with non-zero cross-validation sensitivity (Random Forest, out-of-range imputation for both numerical and categorical features, SMOTE, no tuning). When retrained on the full training set and applied to the hold-out test set (*n* = 563, tICH *n* = 40), it achieved an AUC of 0.517 (95% CI: 0.430–0.604), indicating discrimination close to chance, and a decline of 0.155 from the cross-validation estimate. Sensitivity was 7.5% and specificity 96.0%, with three of 40 tICH cases correctly identified (TP = 3, FN = 37, TN = 502, FP = 21). Complete performance metrics for all 20 configurations are reported in Supplementary Table S3; the operating-point (rule-out) metrics are available for 17 of the 20, as the three tuned SVM configurations lack stored out-of-fold predictions and are reported for discrimination and calibration only. Hold-out AUC ranged from 0.517 to 0.585 across the 20 configurations; at the naïve 0.5 threshold (Figure 4), balanced accuracy was 49.9–51.7% and the Matthews correlation coefficient was near zero throughout.

To assess the stability of this single hold-out estimate, the train–test split was repeated across 200 stratified random partitions (Supplementary Figure S2); the resulting variability is discussed in Section 4.

### 3.5. Center-Specific Analysis

Of the 2250 analytic patients, 1299 were treated in Basel and 951 in Munich. tICH prevalence was 6.3% in Basel and 8.0% in Munich, a difference that did not reach statistical significance (odds ratio 1.29, 95% CI 0.93–1.78; Fisher’s exact *p* = 0.13). The stratified train–test split preserved this distribution, yielding 21 tICH cases among 332 Basel patients and 19 among 231 Munich patients in the hold-out set.

Center-level sample sizes and event counts are given in Supplementary Table S4. Given fewer than 25 tICH events per center in the hold-out set and the absence of any selected model, center-specific operating-point performance cannot be reliably estimated and is therefore not reported; the pooled operating-point analysis for all configurations is given in Supplementary Table S3.

### 3.6. Clinical Characterization of the tICH Cases

Among the 40 tICH cases in the hold-out set, 23 (61%) had a Glasgow Coma Scale of 15 and 35 (92%) a GCS of 14 or 15; only one had documented loss of consciousness, and 34 (85%) had been triaged as Emergency Severity Index 3 or 4. Thirteen (32%) were receiving an oral anticoagulant. Across all 20 top-ranked configurations, these cases received low predicted probabilities (median 0.102), showing that the models assigned the tICH cases low risk regardless of algorithm, preprocessing or class-balancing strategy—consistent with the limited discriminatory signal in the routine pre-CT feature set.

## 4. Discussion

This study systematically benchmarked 1224 machine learning pipeline configurations for the prediction of tICH in 2250 older emergency patients following LEF, using pre-CT clinical features from a bicentric binational cohort. Across the 20 top-ranked configurations, hold-out discrimination remained near-chance (AUC 0.517–0.585), and at a training-derived rule-out operating point (≥95% cross-validation sensitivity) none of the 17 configurations with derivable operating points combined adequate sensitivity with clinically useful specificity (specificity 0–13%). Despite all evaluated algorithms outperforming the featureless baseline, the discrimination and sensitivity achieved are clinically insufficient for deployment as a CT decision-making tool. The consistently near-chance performance across all 1224 pipeline configurations indicates that no algorithm, preprocessing strategy, or tuning configuration overcame it under the present conditions. Rather, the constraint most plausibly arises from a combination of factors intrinsic to this dataset: the limited discriminatory signal of the routinely available pre-CT feature set, the low outcome prevalence (7.0%), and a training sample well below the size required for reliable model development at this event rate. The restriction to CT-imaged patients further limits the range of clinical presentations represented. We therefore interpret the feature set as one contributor among several, rather than as the sole limiting factor. This is in agreement with a recently published editorial emphasizing that statistical performance metrics alone do not establish clinical impact in medical machine learning [20].

The modest performance should be interpreted in light of the characteristics of the present cohort and feature set. First, tICH prevalence is low (7.0%) in older patients following LEF [4,5]. Second, clinical presentation is frequently non-specific: neurological findings are often absent or subtle; further medication effects, comorbidities, and age-related physiological changes might reduce the discriminatory value of individual clinical features [35]. Consistent with this, the tICH cases were predominantly clinically inconspicuous on routine pre-CT parameters—alert (61% with GCS 15), rarely with documented loss of consciousness, and triaged as moderate or low urgency (85% ESI 3 or 4)—precisely the presentations in which routine variables offer little discriminatory signal. After correction for multiple comparisons, statistically significant univariate differences between tICH and non-tICH groups persisted only for GCS, platelet count and loss of consciousness, and even these carried only modest unadjusted effect sizes. A recent systematic review and meta-analysis of 22,520 patients across 17 observational studies reported adjusted ORs (AOR) from multivariable models—a more conservative estimate of independent effect—with only focal neurologic signs (AOR 4.4), external signs of head trauma (AOR 2.7), loss of consciousness (AOR 1.6), and male sex (AOR 1.4) remaining independently associated with tICH after adjustment [36]. The present model included several anticoagulant and antiplatelet exposures (vitamin K antagonists, NOACs, single/dual antiplatelet therapy and LMWH) as candidate predictors. Among these, only LMWH use reached nominal significance in univariate testing (OR 3.56, 95% CI 1.03–10.04, *p* = 0.023), and this did not survive Benjamini–Hochberg correction—the low event rate and correspondingly wide confidence interval mark it as a fragile signal. Consistent with this, anticoagulant use in general was not a significant risk factor in that meta-analysis (OR 0.8, 95% CI 0.7–1.0) [36].

Studies that achieved higher ML performance in comparable settings did so under substantially different conditions. Abe et al. [16] reported a sensitivity of 74% and specificity of 75% in a prehospital triage model for tICH detection, using a cohort of 2123 patients; critically, that model incorporated prehospital vital signs and neurological examination findings measured continuously and prospectively, rather than routine EHR variables, and was not restricted to LEFs and included all age groups. Tunthanathip et al. [37] reported a Naive Bayes model with AUC of 0.70 and sensitivity of 0.97 in elderly TBI patients. But this cohort included a relevant proportion of patients with high energy transfer trauma like traffic accidents, resulting in a tICH-equivalent event rate of 19.6%, nearly three times the prevalence in the present study. This substantially increases the available training signal. These comparisons illustrate that the present results do not imply that ML cannot contribute to tICH risk stratification in general, but rather that pre-CT routine clinical variables as available in this specific retrospective dataset, at this prevalence and sample size, do not provide sufficient discriminatory signal to support a clinically viable model.

Beyond these ML studies, the most directly relevant benchmark for the clinical decision in the older adult with LEF is a non-ML clinical decision rule. The Falls Decision Rule (FDR) was derived specifically to exclude clinically important intracranial bleeding without CT in older adults after ground-level falls [14], and has been externally validated in a comparable population [15]. In that prospective validation of 800 patients, with a tICH prevalence of 6.1%, closely matching the 7.0% observed here, the rule achieved a sensitivity of 97.9% (95% CI 89.1–99.9) and a negative predictive value of 99.5% at a specificity of 31.9%, potentially reducing CT use by approximately one-third. This comparison is not fully like-for-like: the Falls Decision Rule targets clinically important intracranial hemorrhage—bleeds requiring clinical action—whereas the outcome modeled here comprises any traumatic intracranial hemorrhage detected on CT, a broader and more inclusive endpoint. The contrast with our results is instructive: at essentially the same outcome prevalence and in the same clinical population, a rule built from a small number of purpose-selected clinical criteria, including definite exclusion of a head injury based on the patient’s medical history, no amnesia, absence of new neurological abnormalities on clinical examination, and a clinical frailty score of 5 or below, achieves the rule-out sensitivity required for safe CT reduction, whereas the routinely available structured variables evaluated here did not. This is consistent with our central interpretation that the limitation lies substantially in the discriminatory content of the available feature set, and it points to the type of clinical information (e.g., targeted examination findings, beyond the routine administrative and laboratory variables available here) that a future predictive model would likely need to incorporate. We did not present a formal net-benefit analysis. Across the evaluated configurations, the trade-off central to any rule-out use—CT scans avoided versus tICH cases missed—was not favorably resolved, and clinical utility was therefore not established.

This interpretation is further supported by the sample size constraint. Applying the minimum sample size criteria of Riley et al. [38] to our setting (18 predictors, 7% prevalence, and an anticipated AUC of 0.65, chosen as a conservative estimate to avoid underestimating the minimum required sample size) yields an approximate minimum on the order of 8500 patients with 594 events, roughly five times the available training sample of 1687 patients with 118 tICH cases. Because these criteria were derived for the development of a single prediction model rather than for a heterogeneous benchmark of many pipelines, this figure should be read as an approximate, order-of-magnitude benchmark under the stated assumptions rather than as an exact requirement for the present benchmarking exercise. Underpowering at this magnitude is expected to result in overfitting and optimistic cross-validation estimates, as observed in the 0.155-unit CV-to-holdout AUC decline. This is not a problem specific to the present study: a systematic review by Dhiman et al. [39] found that 73% of published prediction model-development studies used sample sizes below the recommended minimum, with a median event deficit of 75 cases. This instability is directly demonstrable in the present data. When the train–test split was repeated across 200 stratified random partitions while holding the model fixed, hold-out AUC ranged from 0.507 to 0.694 (median 0.606)—a spread of nearly 0.19 AUC units attributable solely to which patients happened to fall into the test set. By contrast, varying only the model’s random seed while holding the split fixed produced a spread of just 0.039. The hold-out estimate is therefore governed far more by the partition than by the model, which is the expected behavior at this number of events and underscores that any single hold-out AUC—including the one reported here—should be read as one drawn from a wide distribution rather than as a precise estimate of model performance. Taken together, these considerations indicate that the present work should be interpreted as a systematic exploratory benchmarking study that characterizes the practical limits of prediction in this specific real-world dataset, rather than as a definitive model-development study. Accordingly, these results do not support the use of routine pre-CT clinical variables, in isolation, for automated tICH risk stratification or CT decision-making in this population, and no clinical deployment is warranted on the basis of the present data. Future studies should target substantially larger cohorts, ideally through prospective collection of structured data on multicenter levels. Additionally, it remains an open question whether additional feature domains, such as point-of-care biomarkers, clinical frailty scores, or more structured documentation of clinical examinations especially including impact locations of the signs of head trauma [40] could augment predictive signal beyond routine pre-CT clinical variables.

A critical methodological observation was that, at the default 0.5 threshold, pipelines without class balancing predicted the majority class and therefore yielded zero cross-validation sensitivity, despite competitive CV-AUC values (up to 0.679). This reflects the interaction of the decision threshold with severe class imbalance—an operating-point phenomenon—rather than an intrinsic requirement for resampling to achieve discrimination; the same shift could equivalently be obtained by lowering the classification threshold. It nonetheless illustrates that in severely imbalanced datasets, AUC alone is an insufficient evaluation criterion and that sensitivity at the intended operating point must be examined explicitly for clinical screening applications, consistent with published recommendations on the use of class imbalance corrections in risk prediction. Among pipelines without class balancing, cross-validation sensitivity at the default threshold was consistently zero, whereas resampling—whether by SMOTE or by random oversampling—shifted the operating point so that minority-class cases were detected at that threshold. The accompanying reduction in AUC is expected, as resampling methods do not improve rank-order discrimination but shift the sensitivity–specificity balance toward minority-class detection. The SMOTE-based configurations were moreover poorly calibrated (calibration slope 0.087, well below the ideal of 1.0; intercept −1.05), consistent with the tendency of SMOTE to inflate predicted probabilities for the minority class, which limits the direct clinical interpretability of its probability outputs.

Imputation strategy had negligible impact on performance, with differences across all strategies remaining below 0.02 AUC units, consistent with evidence that imputation method choice has limited downstream influence at low overall missingness rates. Among learners, Random Forest and Elastic Net consistently outperformed more complex methods, a pattern commonly observed in small-to-moderate clinical datasets where complex algorithms require more data to exploit their additional flexibility than was available here. Hyperparameter tuning effects were learner-specific: Elastic Net and Random Forest showed modest improvements, while XGBoost and k-NN showed decreased CV-AUC under tuning, suggesting their default hyperparameters were already better suited to this dataset, in line with the empirically derived observations of Probst et al. [34].

The contribution of this work lies not in a predictive model but in the systematic and reproducible characterization of the performance obtained—and its limits—from routinely available pre-CT variables in this population. By exhaustively evaluating 1224 pipeline configurations rather than reporting a single tuned model, we can state that none of the evaluated pipelines produced clinically adequate performance under the present sample size, cohort selection, feature availability, thresholding, and analytic framework: no combination of algorithm, imputation, class balancing, or hyperparameter tuning overcame this limit. This distinction cannot be drawn from a single-model study, in which a modest result is indistinguishable from an inadequately optimized one. The concern that such negative findings are of limited value overlooks a well-documented asymmetry in the medical machine-learning literature: optimistic single-model reports are published far more readily than rigorous negative results, and reviews of the field have repeatedly found that the large majority of published models are undermined by methodological shortcomings, optimistic evaluation, and limited reproducibility, leaving few, if any, that are clinically usable [41,42]. Against this background, a transparent, fully reproducible benchmark that quantifies its own estimation uncertainty and delineates the limits of a routine feature set is not a null result to be dismissed, but a corrective to the premature optimism these reviews caution against. It defines the performance that future studies incorporating additional data domains must exceed to justify claims of clinical utility, and it does so with methods—a transparent, training-derived operating point, quantified estimation uncertainty, and code available from the authors on reasonable request—that the same literature identifies as necessary and too often absent.

Several limitations apply. First, the hold-out estimates rest on a single, previously inspected partition, which is unstable at this number of events (as shown by the split-variability analysis). Secondly, no external validation was performed, which is required before any clinical translation. Thirdly, the retrospective bicentric design from two European university hospitals provides a clinically relevant dataset that reflects real-life routine tertiary emergency care for older adults following LEF, presenting to the ED without prior trauma team activation. However, this also includes the risk for selection bias and limits applicability to primary or secondary care levels or non-European settings. Importantly, the analytic cohort was restricted to patients who underwent cranial CT, whereas the intended clinical question concerns whether CT should be performed. This CT-based verification means the model was developed in a population already selected for imaging, which may not represent the broader group of older fall patients in whom the imaging decision arises; the results should therefore be interpreted as applying to CT-imaged older adults after LEF rather than to all such patients. Accordingly, the present findings characterize the discriminatory limits of routine pre-CT variables within a CT-imaged population and do not establish the safety of withholding CT in the broader group of older adults after LEF in whom the imaging decision arises. However, the clinical decision to perform CT imaging of the head was based on the Canadian CT Head Rule [10], which was the standard of care in both study centers at the time patients were included in the study. Finally, the laboratory predictors (serum sodium, platelet count, and prothrombin time) were obtained from admission blood sampling and, although typically ordered as standard of care at the time of triage, their results may in individual cases have become available only after the decision to perform CT; their status as strictly pre-decision variables therefore cannot be guaranteed. To probe this uncertainty we repeated the complete benchmarking and ranking procedure after excluding these three laboratory predictors (a 15-feature model); performance remained near-chance (cross-validation AUC of 0.666–0.669, hold-out AUROC of 0.532–0.555; specificity of 6.1–9.2% at the 95% rule-out operating point, 38–39 of 40 tICH cases identified; Supplementary Table S5), so their timing does not affect the conclusion. Lastly, features with high missingness were excluded, which may have removed information where missingness is itself informative (missing-not-at-random)—for example, where an unrecorded finding indicates its absence rather than a missing measurement; reintroducing such features under explicit missingness assumptions is a direction for future work.

## 5. Conclusions

This study presents a systematic exploratory benchmarking of 1224 machine learning pipeline configurations for pre-CT tICH prediction in older adults following LEF, using routinely available clinical features from a well-characterized retrospective bicentric cohort. Despite comprehensive evaluation of diverse algorithms, preprocessing strategies, class-balancing approaches, and hyperparameter settings, hold-out discrimination remained near-chance across all 20 top-ranked configurations (AUC 0.517–0.585); at the naïve 0.5 threshold, an illustrative configuration reached a sensitivity of 7.5%, identifying only three of 40 tICH cases on the hold-out set. These findings suggest that none of the evaluated pipelines produced clinically adequate performance under the present sample size, cohort selection, feature availability, thresholding, and analytic framework; this near-chance performance persisted across all configurations and most plausibly reflects a combination of limited feature signal, low outcome prevalence, and a sample size below the level required for reliable development at this event rate. The relative contribution of each factor cannot be resolved from the present data. Taken together, the present work contributes to the responsible development of clinical machine learning by transparently documenting the limited performance obtained from routine EHR-based features in this real-world ED cohort. Future work should target substantially larger prospective multicenter cohorts, incorporate additional feature domains—including structured documentation of head trauma examination findings, point-of-care biomarkers, and imaging data—and apply rigorous pre-specified validation designs including external validation and decision curve analysis. In the interim, established clinical decision rules, including the Falls Decision Rule specifically developed and validated for older adults after LEF, may remain the appropriate evidence-based framework for CT decision-making in this population.

## Data Availability

The data presented in this study are available on request from the corresponding author. The data are not publicly available due to patient privacy and data protection regulations applicable to retrospective clinical data collected under the approved ethics protocols (EKNZ 2017-01078; EK LMU 17-217). The analysis code, the fixed random seed (20251002), the pinned software environment (renv lock file), and the derived benchmark and operating-point result tables have been deposited in Zenodo (https://doi.org/10.5281/zenodo.21996510). Because the individual-patient data cannot be shared, the per-configuration prediction objects required to re-execute the downstream steps are withheld; the repository provides the code and environment together with the aggregate result tables.

## Supplementary Materials

The following supporting information can be downloaded at https://www.mdpi.com/article/10.3390/diagnostics16172840/s1, Figure S1: Schematic illustration of the cross-validation procedure applied to each pipeline configuration; Figure S2: Variability of hold-out performance across 200 stratified random train–test splits; Table S1: Baseline characteristics of the study cohort and available features (*N* = 2250); Table S2: Hyperparameter search spaces and algorithm-specific default values; Table S3: Complete hold-out performance of all 20 top-ranked pipeline configurations; Table S4: Center-level sample sizes and tICH prevalence; Table S5: Sensitivity analysis excluding the three laboratory predictors (15-feature models); Text S1: Machine learning algorithms.

## Abbreviations

The following abbreviations are used in this manuscript:

AC/AP	Anticoagulation/antiplatelet therapy
AOR	Adjusted odds ratio
AUC	Area under the receiver operating characteristic curve
AUPRC	Area under the precision–recall curve
BP	Blood pressure
CI	Confidence interval
CRP	C-reactive protein
CT	Computed tomography
CV	Cross-validation
CV-AUC	Cross-validation area under the receiver operating characteristic curve
ED	Emergency department
EHR	Electronic health record
EKNZ	Ethics Committee of Northwest and Central Switzerland
EN	Elastic net
EN-CV	Elastic net with cross-validated λ
ESI	Emergency severity index
FDR	Falls decision rule
FN	False negative
FP	False positive
GCS	Glasgow coma scale
HL	Hodges–Lehmann estimator
ICD-10	International Classification of Diseases, 10th Revision
INR	International normalized ratio
IQR	Interquartile range
k-NN	k-nearest neighbors
LEF	Low-energy fall
LMWH	Low-molecular-weight heparin
MCC	Matthews correlation coefficient
MD	Mean difference
ML	Machine learning
NOAC	Non-vitamin K oral anticoagulant
NPV	Negative predictive value
OR	Odds ratio
PPV	Positive predictive value
PTT	Partial thromboplastin time
RAM	Random access memory
RF	Random forest
SD	Standard deviation
Sens	Sensitivity
SMOTE	Synthetic minority oversampling technique
Spec	Specificity
STROBE	Strengthening the reporting of observational studies in epidemiology
SVM	Support vector machine
TBI	Traumatic brain injury
tICH	Traumatic intracranial hemorrhage
TN	True negative
TP	True positive
TPR	True positive rate
VAS	Visual analog scale
VKA	Vitamin K antagonist
XGBoost	Extreme gradient boosting
