# Supplementary material for: Limited Predictability of Traumatic Intracranial Hemorrhage from Routine Pre-CT Clinical Variables in Older Adults with Low-Energy Falls: A Systematic Benchmarking Study in a Retrospective Bicentric Cohort
Source: Diagnostics (Basel). 2026 Sep 3;16(17):2840. doi: 10.3390/diagnostics16172840 (PMC13564560; doi:10.3390/diagnostics16172840)
Supplement: Supplementary file 1 [file diagnostics-16-02840-s001.zip › diagnostics-4382145-supplementary.pdf]

diagnostics

MDPI

Supplementary Text S1: Machine Learning Algorithms

Nine classification algorithms representing diverse learning paradigms — including ensemble methods, kernel-based classifiers, and regularized regression — were evaluated [25-33]:

- Random Forest [25,32] builds a large number of decision trees, each trained on a random subset of the data and features, and combines their predictions by majority vote. This ensemble approach makes it robust to overfitting and capable of capturing complex, non-linear relationships between clinical features and outcomes.
- XGBoost [27] also builds decision trees but sequentially, with each new tree focusing on correcting the errors of the previous ones. Built-in regularization prevents overfitting, making it effective on structured clinical data.
- Support Vector Machine (SVM) [28] identifies the optimal boundary separating classes in a high-dimensional feature space. Two SVM variants with differing hyperparameter search spaces were evaluated (SVM 1 and SVM 2); both explored linear, radial basis function, and polynomial kernels, with SVM 2 additionally including a sigmoid kernel option and a wider cost and gamma search range.
- k-Nearest Neighbors (k-NN) [29] classifies a new patient by looking at the k most similar patients in the training set and assigning the majority outcome. It requires no explicit model training but is sensitive to feature scaling and the choice of k.
- Elastic Net [30,33] is a regularized logistic regression that combines two penalty terms (L1 and L2) to perform automatic feature selection while handling correlated predictors — a common challenge in clinical datasets. A cross-validated variant (Elastic Net CV) was additionally included, which automatically selects the optimal regularization strength.
- Decision Tree [26] classifies patients through a series of binary splits on feature thresholds, producing an interpretable flowchart-like structure. While easy to understand, single decision trees are prone to overfitting and served here primarily as a reference learner.
- Featureless baseline was included as a lower performance bound: it predicts the majority class for every observation regardless of feature values. Any clinically useful model must substantially outperform this baseline.

Supplementary Table S1: Baseline characteristics of the study cohort and available features (*N* = 2250)

Characteristic	*n*	Overall (*N* = 2250)	No tICH (*n* = 2092)	tICH (*n* = 158)	*p*-value	Effect size (95% CI)
Demographics						
**Age [years]** [a)]	2,250	81.8 (8.0)	81.9 (8.0)	81.4 (8.5)	0.567	MD −0.4 (−1.8–1.0)
Missing		*0*	*0*	*0*		
**Sex** [b)]	2,250				**0.020**	OR 1.48 (1.05–2.07)
Female		1448 (64.4%)	1360 (65.0%)	88 (55.7%)		
Male		802 (35.6%)	732 (35.0%)	70 (44.3%)		
Missing		*0*	*0*	*0*		
Clinical and Neurological						
**GCS** [c)]	2070	15 [14–15]	15 [14–15]	15 [14–15]	**<0.001**	HL 0 (0–0) [d)]
Missing		*180 (8%)*	*173 (8.3%)*	*7 (4.4%)*		
**ESI** [b)]	2245				**0.011**	– [e)]
1		18 (0.8%)	15 (0.7%)	3 (1.9%)		
2		249 (11.1%)	221 (10.6%)	28 (17.8%)		
3		1642 (73.1%)	1531 (73.3%)	111 (70.7%)		
4		333 (14.8%)	318 (15.2%)	15 (9.6%)		
5		3 (0.1%)	3 (0.1%)	0 (0.0%)		
Missing		*5 (0.2%)*	*4 (0.2%)*	*1 (0.6%)*		
**Loss of consciousness** [b)]	2250				**0.004**	OR 2.16 (1.27–3.52)
No		2082 (92.5%)	1946 (93.0%)	136 (86.1%)		
Yes		168 (7.5%)	146 (7.0%)	22 (13.9%)		
Missing		*0*	*0*	*0*		
**Mechanism of fall** [b)]	2250				0.394	– [e)]
Fall ≤1 m		88 (3.9%)	82 (3.9%)	6 (3.8%)		
Fall from standing height		1934 (85.9%)	1793 (85.7%)	141 (89.2%)		
Fall from furniture/bed		228 (10.1%)	217 (10.4%)	11 (7.0%)		
Missing		*0*	*0*	*0*		
Symptoms of concussion [b)]	1649				0.016	OR 1.62 (1.09–2.42)
No		1060 (64.3%)	998 (65.1%)	62 (53.4%)		
Yes		589 (35.7%)	535 (34.9%)	54 (46.6%)		
Missing		*601 (26.7%)*	*559 (26.7%)*	*42 (26.6%)*		
**Supraclavicular injury signs** [b)]	2250				0.069	OR 1.35 (0.96–1.90)
No		1211 (53.8%)	1137 (54.3%)	74 (46.8%)		
Yes		1039 (46.2%)	955 (45.7%)	84 (53.2%)		
Missing		*0*	*0*	*0*		

Vital Signs						
Heart rate [bpm] [a)]	1236	80.8 (17.2)	80.7 (17.2)	83.1 (16.6)	0.206	MD +2.5 (−1.4–6.3)
Missing		*1014 (45.1%)*	*936 (44.7%)*	*78 (49.4%)*		
Systolic BP [mmHg] [a)]	1245	143.3 (26.2)	143.2 (26.3)	144.4 (24.7)	0.668	MD +1.2 (−4.4–6.9)
Missing		*1005 (44.7%)*	*928 (44.4%)*	*77 (48.7%)*		
Diastolic BP [mmHg] [a)]	1240	75.6 (14.9)	75.5 (15.0)	76.2 (13.2)	0.641	MD +0.7 (−2.3–3.8)
Missing		*1010 (44.9%)*	*933 (44.6%)*	*77 (48.7%)*		
O_2 saturation [%] [a)]	1207	96.2 (4.8)	96.2 (4.9)	96.1 (2.3)	0.913	MD 0.0 (−0.6–0.6)
Missing		*1043 (46.4%)*	*963 (46.0%)*	*80 (50.6%)*		
Temperature [°C] [c)]	631	36.6 [36.2–37.0]	36.6 [36.2–37.0]	36.4 [35.8–37.1]	0.178	HL −0.2 (−0.4–0.1)
Missing		*1619 (72.0%)*	*1503 (71.8%)*	*116 (73.4%)*		
Respiratory rate [c)]	443	16 [14–20]	16 [14–20]	18 [13–20]	0.584	HL +0 (−1–2)
Missing		*1807 (80.3%)*	*1674 (80.0%)*	*133 (84.2%)*		
Pain score [VAS] [c)]	578	1 [0–4]	1 [0–4]	0 [0–3]	0.576	HL 0 (0–0) [d)]
Missing		*1672 (74.3%)*	*1551 (74.1%)*	*121 (76.6%)*		
Medication						
**No AC/AP** [b)]	2250				0.176	OR 1.26 (0.90–1.77)
No		1372 (61.0%)	1284 (61.4%)	88 (55.7%)		
Yes		878 (39.0%)	808 (38.6%)	70 (44.3%)		
Missing		*0*	*0*	*0*		
**VKA** [b)]	2250				>0.999	OR 1.00 (0.58–1.66)
No		1980 (88.0%)	1841 (88.0%)	139 (88.0%)		
Yes		270 (12.0%)	251 (12.0%)	19 (12.0%)		
Missing		*0*	*0*	*0*		
**NOAC** [b)]	2250				0.899	OR 0.94 (0.53–1.58)
No		1981 (88.0%)	1841 (88.0%)	140 (88.6%)		
Yes		269 (12.0%)	251 (12.0%)	18 (11.4%)		
Missing		*0*	*0*	*0*		
**Single AP** [b)]	2250				0.726	OR 0.93 (0.64–1.32)
No		1503 (66.8%)	1395 (66.7%)	108 (68.4%)		
Yes		747 (33.2%)	697 (33.3%)	50 (31.6%)		
Missing		*0*	*0*	*0*		
**Dual AP** [b)]	2250				>0.999	OR 0.69 (0.08–2.73)
No		2210 (98.2%)	2054 (98.2%)	156 (98.7%)		
Yes		40 (1.8%)	38 (1.8%)	2 (1.3%)		
Missing		*0*	*0*	*0*		
**LMWH** [b)]	2250				**0.023**	OR 3.56 (1.03–10.04)

No		2226 (98.9%)	2073 (99.1%)	153 (96.8%)		
Yes		24 (1.1%)	19 (0.9%)	5 (3.2%)		
Missing		*0*	*0*	*0*		
**AC/AP unknown [b)]**	2250				**0.040**	OR 0.17 (0.00–0.99)
No		2173 (96.6%)	2016 (96.4%)	157 (99.4%)		
Yes		77 (3.4%)	76 (3.6%)	1 (0.6%)		
Missing		*0*	*0*	*0*		
**Polymedication [b)]**	2250				0.535	OR 0.89 (0.61–1.28)
No		1543 (68.6%)	1431 (68.4%)	112 (70.9%)		
Yes		707 (31.4%)	661 (31.6%)	46 (29.1%)		
Missing		*0*	*0*	*0*		
Laboratory Values						
**Serum sodium [mmol/L] [a)]**	2073	137.4 (4.9)	137.4 (4.9)	137.1 (4.7)	0.488	MD −0.3 (−1.1–0.5)
Missing		*177 (7.9%)*	*173 (8.3%)*	*4 (2.5%)*		
**Platelets [10^3/µL] [a)]**	2068	234.7 (83.1)	236.6 (82.8)	211.2 (83.3)	**<0.001**	MD −25.4 (−39.2–−11.6)
Missing		*182 (8.1%)*	*177 (8.5%)*	*5 (3.2%)*		
**Quick [%] [c)]**	2055	89 [74–101]	89 [74–101]	88 [71–100]	0.647	HL −1 (−4–3)
Missing		*195 (8.7%)*	*190 (9.1%)*	*5 (3.2%)*		
Serum albumin [g/L] [a)]	1307	29.6 (10.6)	30.1 (10.0)	24.0 (14.2)	<0.001	MD −6.2 (−8.9–−3.4)
Missing		*943 (41.9%)*	*897 (42.9%)*	*46 (29.1%)*		
Serum urea [mmol/L] [c)]	1387	6.6 [5.1–9.1]	6.7 [5.1–9.2]	6.3 [4.9–8.4]	0.243	HL −0.3 (−0.8–0.2)
Missing		*863 (38.4%)*	*825 (39.4%)*	*38 (24.1%)*		
Hemoglobin [g/L] [a)]	1147	127.7 (18.4)	127.8 (18.3)	126.1 (20.4)	0.482	MD −1.7 (−6.4–3.0)
Missing		*1103 (49.0%)*	*1023 (48.9%)*	*80 (50.6%)*		
CRP [mg/L] [c)]	1138	4.9 [1.7–15.7]	4.9 [1.7–15.9]	4.6 [1.3–13.8]	0.528	HL −0.3 (−1.4–0.7)
Missing		*1112 (49.4%)*	*1032 (49.3%)*	*80 (50.6%)*		
Serum creatinine [mg/dL] [a)]	921	1.22 (0.71)	1.22 (0.68)	1.33 (1.02)	0.332	MD +0.12 (−0.12–0.36)
Missing		*1329 (59.1%)*	*1246 (59.6%)*	*83 (52.5%)*		
INR [c)]	916	1.00 [0.98–1.10]	1.00 [0.90–1.10]	1.00 [1.00–1.10]	0.115	HL +0.00 (0–0.10)
Missing		*1334 (59.3%)*	*1251 (59.8%)*	*83 (52.5%)*		
PTT [sec] [c)]	915	24 [22–27]	24 [22–27]	24 [22–27]	0.851	HL 0 (−1–1)
Missing		*1335 (59.3%)*	*1252 (59.8%)*	*83 (52.5%)*		
Serum S100 [µg/L] [a)]	695	0.51 (0.85)	0.49 (0.82)	0.78 (1.21)	0.094	MD +0.29 (−0.05–0.63)
Missing		*1555 (69.1%)*	*1450 (69.3%)*	*105 (66.5%)*		

Data presented as mean (SD), median [IQR], or *n* (%). Missing values are reported as *n* (% of respective group)

Statistical tests: [a] Welch two-sample t-test; [b] Fisher's exact test; [c] Wilcoxon rank-sum test. Effect sizes (direction: tICH minus No tICH): MD = mean difference with 95% CI (Welch's formula); OR = odds ratio with 95% CI (Fisher's exact); HL = Hodges–Lehmann estimator with 95% CI.
[d] Hodges–Lehmann CI collapses to zero due to near-identical distributions at the median level. [e] Multinomial variable: overall Fisher's *p*-value reported; group-level OR not applicable.
Feature names in bold indicate inclusion in the final feature set for machine learning model development (selected based on clinical availability prior to CT decision and <10% missing values in the total cohort). *p*-values in bold indicate statistically significant differences between tICH and No tICH groups ($p < 0.05$). Multiplicity: with 34 univariate comparisons, only GCS, platelet count, serum albumin and loss of consciousness remain significant after Benjamini–Hochberg false-discovery-rate correction ($q < 0.05$); the remaining nominal associations ($p < 0.05$) do not survive correction and may reflect chance.
Abbreviations: AC/AP, anticoagulation/antiplatelet therapy; BP, blood pressure; CRP, C-reactive protein; ESI, Emergency Severity Index; GCS, Glasgow Coma Scale; HL, Hodges–Lehmann estimator; INR, international normalized ratio; LMWH, low-molecular-weight heparin; MD, mean difference; NOAC, non-vitamin K oral anticoagulant; OR, odds ratio; PTT, partial thromboplastin time; tICH, traumatic intracranial hemorrhage; VAS, visual analog scale; VKA, vitamin K antagonist.

Supplementary Table S2: Hyperparameter search spaces and algorithm-specific default values

Learner	Parameter	Search Space	Default (No Tuning)
Random Forest	num.trees	100–2000	500
	mtry (ratio → count)	0.05–1.0 (→ $\lfloor \text{ratio} \times p \rfloor$)	$\lfloor \sqrt{p} \rfloor$
	sample.fraction	0.1–1.0	1.0
	min.node.size	trafo: nrows^x, $x \in [0,1]$	1
	replace	TRUE / FALSE	TRUE
	respect.unordered.factors	ignore / order / partition	ignore
XGBoost	nrounds	50–1500	1000 †
	eta	2^{-7}–2^{-1}	0.3
	max_depth	2–12	6
	subsample	0.5–1.0	1.0
	colsample_bytree	0.5–1.0	1.0
	colsample_bylevel	0.5–1.0	1.0
	min_child_weight	2^{0}–2^{7}	1
	alpha (L1)	2^{-10}–2^{10}	0
	lambda (L2)	2^{-10}–2^{10}	1
SVM 1§	kernel	radial, linear, polynomial	radial
	cost (C)	2^{-5}–2^{8}	1
	gamma ‡	2^{-10}–2^{3}	$1/p$
	degree ‡	2–5	3
SVM 2§	kernel	radial, linear, polynomial, sigmoid	radial
	cost (C)	2^{-10}–2^{10}	1
	gamma ‡	2^{-10}–2^{10}	$1/p$
	degree ‡	2–5	3
Decision Tree	cp	0–1	0.01
	maxdepth	1–30	30
	minsplit	1–60	20
	minbucket	1–60	7
k-NN	k	1–30	7
Elastic Net	alpha	0–1	1 (Lasso)
	lambda	2^{-10}–2^{10}	0.01
Elastic Net (CV)	alpha	0–1	1 (Lasso)
	lambda	internal CV only	λ.1se

Hyperparameter search spaces were defined following Probst et al. [34]. For each algorithm, tuned parameters, their search ranges, and the default values used in the "no tuning" condition (as implemented in mlr3) are listed above. Tuning employed 30 random search iterations optimizing CV-AUC. p = number of features after preprocessing; nrows = number of training observations; x = sampled exponent, $x \in [0,1]$; $\lfloor \cdot \rfloor$ denotes the floor function (rounding down to the nearest integer); → denotes transformation applied after sampling: the sampled ratio is converted to an integer feature count via $\lfloor \text{ratio} \times p \rfloor$ prior to model fitting. † nrounds has

no default in the xgboost package itself; mlr3learners applies an internal default of nrounds = 1000. ‡ Kernel-specific parameters (gamma, degree) were excluded from the search space when the linear kernel was selected. § SVM 1 and SVM 2 differ in their hyperparameter search spaces: SVM 1 uses a narrower cost range (2^{-5}–2^{8}) and gamma range (2^{-10}–2^{3}); SVM 2 uses wider ranges (cost: 2^{-10}–2^{10}, gamma: 2^{-10}–2^{10}) and additionally includes a sigmoid kernel option. λ.1se: the largest λ value within one standard error of the minimum cross-validation error, as implemented in glmnet.

diagnostics

MDPI

Supplementary Table S3: Complete hold-out performance of all 20 top-ranked pipeline configurations (*n* = 563; 40 tICH cases). For each of the 17 configurations with stored out-of-fold predictions the two rows correspond to the two training-derived rule-out thresholds (≥95% and ≥97.9% cross-validation sensitivity); threshold-independent metrics are shown once, spanning both rows. The three tuned SVM configurations lack stored out-of-fold predictions, so only their discrimination and calibration are reported

Configuration	Rule-out	AUROC (95% CI)	AUPRC	Cal. slope	Cal. int.	Brier	TP	FN	TN	FP	Sens. %	Spec. %	NPV %	PPV %	F1	MCC	Bal. acc. %	CT avd. %
RF (mean/none)	≥95 %	0.552 (0.465–0.639)	0.106	0.269	−0.09	0.069	39	1	63	460	97.5	12.0	98.4	7.8	0.145	0.077	54.8	11.4
	≥97.9 %						40	0	49	474	100.0	9.4	100.0	7.8	0.144	0.085	54.7	8.7
RF (mean/mode)	≥95 %	0.553 (0.466–0.639)	0.106	0.270	−0.09	0.069	39	1	64	459	97.5	12.2	98.5	7.8	0.145	0.078	54.9	11.5
	≥97.9 %						40	0	50	473	100.0	9.6	100.0	7.8	0.145	0.086	54.8	8.9
RF (mean/oor)	≥95 %	0.552 (0.465–0.639)	0.106	0.269	−0.09	0.069	39	1	63	460	97.5	12.0	98.4	7.8	0.145	0.077	54.8	11.4
	≥97.9 %						40	0	49	474	100.0	9.4	100.0	7.8	0.144	0.085	54.7	8.7
RF (mean/sample)	≥95 %	0.553 (0.466–0.639)	0.106	0.270	−0.09	0.069	39	1	64	459	97.5	12.2	98.5	7.8	0.145	0.078	54.9	11.5
	≥97.9 %						40	0	50	473	100.0	9.6	100.0	7.8	0.145	0.086	54.8	8.9
RF (median/none)	≥95 %	0.550 (0.468–0.631)	0.116	0.262	−0.09	0.069	39	1	67	456	97.5	12.8	98.5	7.9	0.146	0.081	55.2	12.1
	≥97.9 %						40	0	43	480	100.0	8.2	100.0	7.7	0.143	0.080	54.1	7.6
RF (median/mode)	≥95 %	0.550 (0.469–0.631)	0.116	0.263	−0.09	0.069	39	1	68	455	97.5	13.0	98.6	7.9	0.146	0.082	55.3	12.3
	≥97.9 %						40	0	43	480	100.0	8.2	100.0	7.7	0.143	0.080	54.1	7.6
RF (median/oor)	≥95 %	0.550 (0.468–0.631)	0.116	0.262	−0.09	0.069	39	1	67	456	97.5	12.8	98.5	7.9	0.146	0.081	55.2	12.1
	≥97.9 %						40	0	43	480	100.0	8.2	100.0	7.7	0.143	0.080	54.1	7.6
RF (median/sample)	≥95 %	0.550 (0.469–0.631)	0.116	0.263	−0.09	0.069	39	1	68	455	97.5	13.0	98.6	7.9	0.146	0.082	55.3	12.3
	≥97.9 %						40	0	43	480	100.0	8.2	100.0	7.7	0.143	0.080	54.1	7.6
RF (oor/mode, SMOTE)	≥95 %	0.517 (0.429–0.604)	0.077	0.087	−1.05	0.093	38	2	61	462	95.0	11.7	96.8	7.6	0.141	0.054	53.3	11.2
	≥97.9 %						39	1	31	492	97.5	5.9	96.9	7.3	0.137	0.038	51.7	5.7
RF (oor/oor, SMOTE)	≥95 %	0.517 (0.430–0.604)	0.077	0.087	−1.05	0.093	38	2	61	462	95.0	11.7	96.8	7.6	0.141	0.054	53.3	11.2
	≥97.9 %						39	1	31	492	97.5	5.9	96.9	7.3	0.137	0.038	51.7	5.7

Configuration	Rule-out	AUROC (95% CI)	AUPRC	Cal. slope	Cal. int.	Brier	TP	FN	TN	FP	Sens. %	Spec. %	NPV %	PPV %	F1	MCC	Bal. acc. %	CT avd. %
RF (oor/sample, SMOTE)	≥95 %	0.517 (0.429–0.604)	0.077	0.087	−1.05	0.093	38	2	61	462	95.0	11.7	96.8	7.6	0.141	0.054	53.3	11.2
	≥97.9 %						39	1	31	492	97.5	5.9	96.9	7.3	0.137	0.038	51.7	5.7
RF (mean/mode, SMOTE, tuned)	≥95 %	0.585 (0.498–0.671)	0.088	0.370	−1.88	0.137	40	0	0	523	100.0	0.0	—	7.1	0.133	—	50.0	0.0
	≥97.9 %						40	0	0	523	100.0	0.0	—	7.1	0.133	—	50.0	0.0
RF (mean/oor, SMOTE, tuned)	≥95 %	0.585 (0.498–0.671)	0.088	0.370	−1.88	0.137	40	0	0	523	100.0	0.0	—	7.1	0.133	—	50.0	0.0
	≥97.9 %						40	0	0	523	100.0	0.0	—	7.1	0.133	—	50.0	0.0
RF (mean/sample, SMOTE, tuned)	≥95 %	0.585 (0.498–0.671)	0.088	0.370	−1.88	0.137	40	0	0	523	100.0	0.0	—	7.1	0.133	—	50.0	0.0
	≥97.9 %						40	0	0	523	100.0	0.0	—	7.1	0.133	—	50.0	0.0
SVM (median/mode, oversampling, tuned)	—	0.571 (0.476–0.665)	0.099	0.189	−2.67	0.227	—	—	—	—	—	—	—	—	—	—	—	—
SVM (median/oor, oversampling, tuned)	—	0.571 (0.476–0.665)	0.099	0.189	−2.67	0.227	—	—	—	—	—	—	—	—	—	—	—	—
SVM (median/sample, oversampling, tuned)	—	0.571 (0.476–0.665)	0.099	0.189	−2.67	0.227	—	—	—	—	—	—	—	—	—	—	—	—
EN (mean/mode)	≥95 %	0.561 (0.467–0.655)	0.093	0.395	0.00	0.067	40	0	12	511	100.0	2.3	100.0	7.3	0.135	0.041	51.1	2.1
	≥97.9 %						40	0	1	522	100.0	0.2	100.0	7.1	0.133	0.012	50.1	0.2
EN (mean/oor)	≥95 %	0.561 (0.467–0.655)	0.093	0.395	0.00	0.067	40	0	12	511	100.0	2.3	100.0	7.3	0.135	0.041	51.1	2.1
	≥97.9 %						40	0	1	522	100.0	0.2	100.0	7.1	0.133	0.012	50.1	0.2
EN (mean/sample)	≥95 %	0.561 (0.467–0.655)	0.093	0.395	0.00	0.067	40	0	12	511	100.0	2.3	100.0	7.3	0.135	0.041	51.1	2.1
	≥97.9 %						40	0	1	522	100.0	0.2	100.0	7.1	0.133	0.012	50.1	0.2

Hold-out performance (n = 563; 40 tICH). AUROC, AUPRC, calibration slope/intercept and Brier are threshold-independent. For each configuration the training-derived threshold reaching ≥95% (upper row) and ≥97.9% (lower row, matched to the sensitivity reported for the Falls Decision Rule, which targets clinically important ICH rather than any tICH) cross-validation sensitivity was applied to the hold-out. TP/FN/TN/FP = true/false positives/negatives; Sens = sensitivity; Spec = specificity; NPV/PPV = negative/positive predictive value; MCC = Matthews correlation coefficient; Bal. acc. = balanced accuracy; CT avd. = proportion of CT examinations potentially avoided. The three tuned SVM configurations have no stored cross-validation predictions, so only their (threshold-independent) discrimination and calibration are reported. AUROC = area under the receiver-operating-characteristic curve; AUPRC = area under the precision–recall curve; RF = random forest; EN = elastic net.

diagnostics

Supplementary Table S4: Center-level sample sizes and tICH prevalence.

Center	Cohort *n*	Cohort tICH (%)	Hold-out *n*	Hold-out tICH
Basel	1299	82 (6.3%)	332	21
Munich	951	76 (8.0%)	231	19
Total	2250	158 (7.0%)	563	40

Center-level sample sizes and tICH counts for the full analytic cohort and the hold-out set; percentages are the within-center tICH prevalence. No performance metrics are shown; per-center operating-point performance is not reported (see Section 3.5).

Supplementary Table S5. Sensitivity analysis excluding the three laboratory predictors (serum sodium, platelet count, prothrombin time): 15-feature performance of the 20 top-ranked configurations of the recomputed ranking at both training-derived rule-out operating points (hold-out set, *n* = 563; 40 tICH cases).

Configuration	Rule-out	CV-AUC	Hold-out AUROC	Sens. %	Spec. %	NPV %	PPV %	tICH found /40	CT avd. %
EN (median/mode, over)	≥95 %	0.669	0.555	95.0	9.2	96.0	7.4	38	8.9
	≥97.9 %			100.0	5.2	100.0	7.5	40	4.8
EN (mean/mode, over)	≥95 %	0.669	0.555	95.0	9.2	96.0	7.4	38	8.9
	≥97.9 %			100.0	5.2	100.0	7.5	40	4.8
EN (median/oor, over)	≥95 %	0.669	0.555	95.0	9.2	96.0	7.4	38	8.9
	≥97.9 %			100.0	5.2	100.0	7.5	40	4.8
EN (mean/oor, over)	≥95 %	0.669	0.555	95.0	9.2	96.0	7.4	38	8.9
	≥97.9 %			100.0	5.2	100.0	7.5	40	4.8
EN (median/sample, over)	≥95 %	0.669	0.555	95.0	9.2	96.0	7.4	38	8.9
	≥97.9 %			100.0	5.2	100.0	7.5	40	4.8
EN (mean/sample, over)	≥95 %	0.669	0.555	95.0	9.2	96.0	7.4	38	8.9
	≥97.9 %			100.0	5.2	100.0	7.5	40	4.8
EN (median/oor)	≥95 %	0.669	0.532	95.0	6.9	94.7	7.2	38	6.7
	≥97.9 %			95.0	6.9	94.7	7.2	38	6.7
EN (mean/oor)	≥95 %	0.669	0.532	95.0	6.9	94.7	7.2	38	6.7
	≥97.9 %			95.0	6.9	94.7	7.2	38	6.7
EN (median/sample)	≥95 %	0.669	0.532	95.0	6.9	94.7	7.2	38	6.7
	≥97.9 %			95.0	6.9	94.7	7.2	38	6.7
EN (mean/sample)	≥95 %	0.669	0.532	95.0	6.9	94.7	7.2	38	6.7
	≥97.9 %			95.0	6.9	94.7	7.2	38	6.7
EN (median/mode)	≥95 %	0.669	0.532	95.0	6.9	94.7	7.2	38	6.7
	≥97.9 %			95.0	6.9	94.7	7.2	38	6.7
EN (mean/mode)	≥95 %	0.669	0.532	95.0	6.9	94.7	7.2	38	6.7
	≥97.9 %			95.0	6.9	94.7	7.2	38	6.7
EN-CV (median/mode, over)	≥95 %	0.667	0.551	97.5	6.1	97.0	7.4	39	5.9
	≥97.9 %			100.0	3.1	100.0	7.3	40	2.8
EN-CV (mean/mode, over)	≥95 %	0.667	0.551	97.5	6.1	97.0	7.4	39	5.9
	≥97.9 %			100.0	3.1	100.0	7.3	40	2.8
EN-CV (median/oor, over)	≥95 %	0.667	0.551	97.5	6.1	97.0	7.4	39	5.9
	≥97.9 %			100.0	3.1	100.0	7.3	40	2.8
EN-CV (mean/oor, over)	≥95 %	0.667	0.551	97.5	6.1	97.0	7.4	39	5.9
	≥97.9 %			100.0	3.1	100.0	7.3	40	2.8
EN-CV (median/sample, over)	≥95 %	0.667	0.551	97.5	6.1	97.0	7.4	39	5.9

	≥97.9 %			100.0	3.1	100.0	7.3	40	2.8
EN-CV (mean/sample, over)	≥95 %	0.667	0.551	97.5	6.1	97.0	7.4	39	5.9
	≥97.9 %			100.0	3.1	100.0	7.3	40	2.8
RF (median/none, under)	≥95 %	0.666	0.548	95.0	7.1	94.9	7.3	38	6.9
	≥97.9 %			97.5	4.6	96.0	7.2	39	4.4
RF (mean/none, under)	≥95 %	0.666	0.548	95.0	7.1	94.9	7.3	38	6.9
	≥97.9 %			97.5	4.6	96.0	7.2	39	4.4

Performance after removing serum sodium, platelet count and prothrombin time (15 of the original 18 features). The entire benchmarking and ranking procedure was repeated on the 15-feature set (all 1656 constructed / 1224 valid configurations; identical stratified 75/25 split and 10-fold cross-validation, seed 20251002), and the 20 configurations with the highest 15-feature cross-validated AUC were selected from the resulting ranking; the configurations shown here therefore differ from those of the primary analysis, as expected when the feature set changes and the entire ranking is recomputed. For each configuration the two rows correspond to the training-derived thresholds reaching ≥95% (upper row) and ≥97.9% (lower row, matched to the Falls Decision Rule) cross-validation sensitivity, applied unchanged to the hold-out; CV-AUC and hold-out AUROC are threshold-independent and are therefore shown once, spanning both rows. Because the score distribution is coarse at this event rate, the sensitivity actually attained on the hold-out at the second threshold ranges from 95.0% to 100%. A reproducibility control recomputing the cross-validated AUC of every deterministic configuration matched the stored benchmark bit-for-bit (maximum $|\Delta| = 0.0000$). Across the top-20, cross-validated AUC (0.666–0.669) and hold-out AUROC (0.532–0.555) remained near chance, specificity at the rule-out point stayed between 6.1% and 9.2% at 95% sensitivity and between 3.1% and 6.9% at the 97.9% threshold, 38–40 of 40 tICH cases were identified, and at most 8.9% of CT examinations could potentially have been avoided. Excluding the potentially post-decision laboratory predictors therefore did not change any conclusion. CV = cross-validation; AUROC = area under the receiver-operating-characteristic curve; NPV/PPV = negative/positive predictive value; CT avd. = proportion of CT examinations potentially avoided; RF = random forest; EN = elastic net; EN-CV = elastic net with cross-validated λ (cv.glmnet); "over" = over-sampling, "under" = under-sampling.

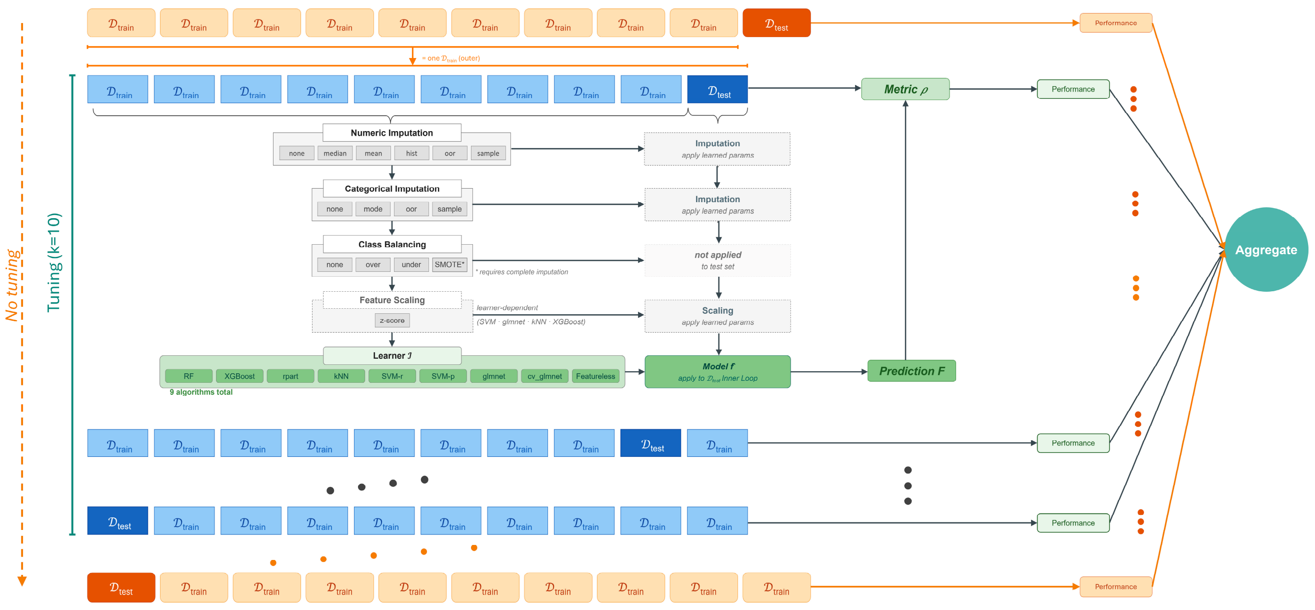

Supplementary Figure S1. Schematic illustration of the cross-validation procedure applied to each pipeline configuration.

The outer 10-fold cross-validation loop (orange) partitions the training set into nine training folds (D_{train}) and one test fold (D_{test}) per iteration, rotating until each fold has served as the test fold once. For pipeline configurations with hyperparameter tuning (Tuning, $k = 10$), an additional inner cross-validation loop is nested within each outer training fold to optimize hyperparameters via random search before applying the model to the outer test fold. For configurations without tuning (No Tuning, blue), algorithm-specific default hyperparameters are used and the model is applied directly to the outer test fold. Preprocessing parameters (imputation statistics, scaling parameters) are estimated on the outer training folds and applied without re-estimation to the outer test fold; class balancing is applied to training folds only. Performance metrics from all outer folds are aggregated to yield the final CV-AUC per pipeline configuration. CV = cross-validation; AUC = area under the receiver operating characteristic curve; D_{train} = training partition; D_{test} = test partition.

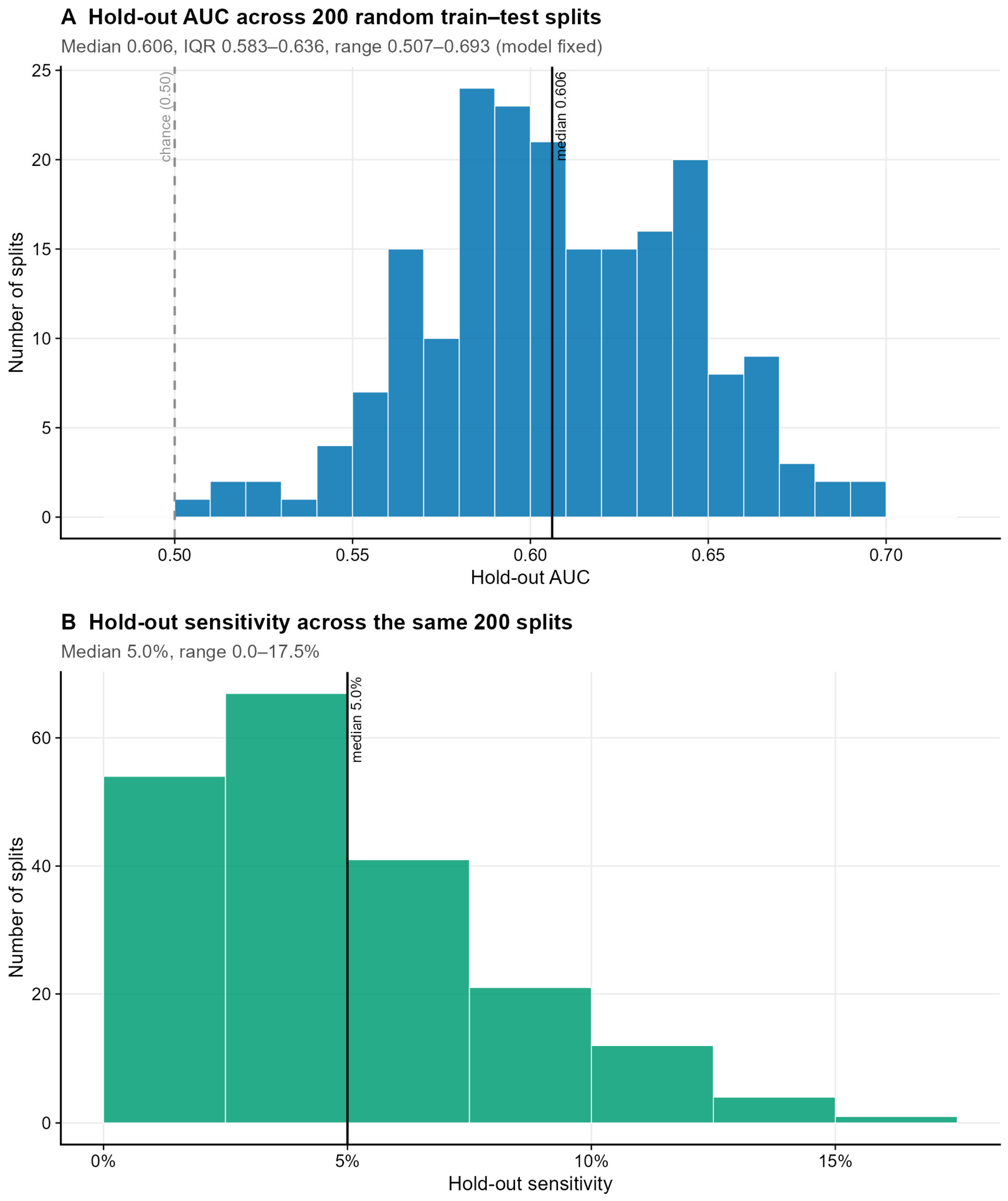

Supplementary Figure S2. Variability of hold-out performance across 200 stratified random train–test splits, with the model held fixed. (A) Hold-out AUC ranged from 0.507 to 0.694 (median 0.606); the dashed line marks chance-level performance (AUC 0.50). For comparison, varying only the model's random seed with the split held fixed produced a far narrower range (span 0.039; reported in the Discussion), indicating that the hold-out estimate is governed primarily by the partition rather than the model. (B) Hold-out sensitivity across the same splits ranged from 0% to 17.5% (median 5.0%). These distributions illustrate the instability of any single hold-out estimate at this number of events (n = 40).
